# Electrocardiogram Interpretation Competency of Medical Interns in Saudi Arabia: A Cross-Sectional Study

**DOI:** 10.7759/cureus.37557

**Published:** 2023-04-14

**Authors:** Ali M Al Mousa, Fatimah M Alhubail, Mohannad Almulhim, Baneen A AlBeladi, Nasser A Almulhim, Abdullah A Almulhim, Ibtisam A Algouf, Abdul Sattar Khan

**Affiliations:** 1 Internal Medicine Department, College of Medicine, King Faisal University, Alahsa, SAU; 2 Clinical Pharmacy Department, College of Clinical Pharmacy, King Faisal University, Alahsa, SAU; 3 Family and Community Medicine Department, College of Medicine, King Faisal University, Alahsa, SAU

**Keywords:** saudi arabia, competency, cardiology, medical intern, electrocardiogram interpretation

## Abstract

Objective

To determine the competence of medical interns in Saudi Arabia in interpreting common ECG abnormalities, explore limitations, and facilitate solutions to the development of ECG interpretation skills in Saudi Arabia.

Methods

This cross-sectional study was conducted from 11 June 2022 to 3 November 2022 using the convenience stratified sampling technique among 373 medical interns (54.4% male and 45.6% female) in 15 medical colleges within Saudi Arabia.

Results

Almost all (91.7%) of the participants recognized the basic ECG elements, as they correctly identified normal ECG patterns. The most well-understood ECG pathologies were ventricular fibrillation, atrial fibrillation, and acute myocardial infarction, which were accurately interpreted by 69.2%, 67.8%, and 61.9% of the participants, respectively. The least understood ECG result was a pathological Q wave, which only 20.9% recognized. Most (63.5%) participants attributed their challenges in ECG interpretation to their inadequate training in college, and 57.4% of them stated that practical case-based training could best facilitate the improvement of their skills in ECG interpretation.

Conclusion

Most of the participants showed unsatisfactory performance in ECG interpretation. Despite their completion of advanced cardiac life support courses, their overall performance did not improve significantly. Most of them believed that their colleges did not adequately train them to read ECGs. Thus, a majority think case-based training is a key strategy for improving their ECG interpretation skills.

## Introduction

Cardiovascular disease (CVD) is the main cause of death globally, accounting for 32% of all mortality [1]. It is now a serious health problem in Gulf Council countries, such as Saudi Arabia, where it is thought to be responsible for about 45.7% of all deaths [2]. Many life-threatening heart diseases are detected using an electrocardiogram (ECG) [3,4]. Thus, ECG interpretation is a crucial skill for undergraduate and postgraduate medical students [5]. Accurate ECG interpretation has been proven to be a determinant of appropriate treatment for many acute cardiovascular emergencies including acute myocardial infarction, arrhythmias, and patients with cardiac arrest [6]. When ECGs are interpreted incorrectly, they can have catastrophic consequences, and patient management plans may become inappropriate [7]. To demonstrate adequate ECG knowledge, it is important to understand the basic mechanisms of some ECG abnormalities [8].

Several studies have been published worldwide to assess the ECG interpretation competency of physicians and medical students. A meta-analysis of 78 studies was published in 2020 to assess the accuracy of the ECG interpretation of physicians and medical students, and it showed low accuracy in the absence of progressive training [9]. Another study carried out among medical students in different medical schools in Poland demonstrated their good level of recognition of primary ECG parameters but low skills in identifying common heart emergencies [10]. A further study conducted in Taif City showed a moderate level of ECG misinterpretation in sixth-year medical students and interns and highlighted the huge impact of ECG courses and self-education on enhancing ECG interpretation skills [11]. However, not enough data are available to estimate the skills of medical interns in Saudi Arabia in recognizing common ECG abnormalities. This study was designed to estimate the skills of medical interns in Saudi Arabia in ECG interpretation.

## Materials and methods

This cross-sectional study was conducted from the 11th of June to the 3rd of November 2022 by using a stratified sampling technique in 15 medical schools around Saudi Arabia. With respect to the aim of the study, an electronic Google Form survey was distributed through social media to assess the medical intern’s competency level in interpreting common ECG abnormalities and to explore limitations and facilitations to develop adequate ECG interpretation skills. The distribution of the survey was facilitated with the aid of 24 data collectors from different universities to ensure the presence of enough participants to represent the population.

The targeted population included medical graduates from Saudi universities who worked at that time as interns. Any individuals who were not interns, didn’t complete the survey, or refused to participate in the study, were excluded. A previously adapted research questionnaire was used to execute the study objectives [12-13]. The questionnaire consisted of four essential divisions: (A) five questions regarding the participants' characteristics and demographic data, (B) five questions about previous enrollment in extracurricular courses related to ECG, (C) 12 multiple-choice questions that included two theoretical questions and 10 clinical questions with ECG records, (D) two additional questions to determine the interns’ perspective of ECG learning barriers. Table 1 demonstrates the questions included in the survey as a tool of assessment.

**Table 1:** Content of the questions in the questionnaire

Item	Subject
Theoretical ECG Q1	Waves and ECG intervals
Theoretical ECG Q2	P wave
Clinical ECG Q1	Atrial flutter
Clinical ECG Q2	Ventricular fibrillation
Clinical ECG Q3	Atrial fibrillation
Clinical ECG Q4	Pathological Q wave
Clinical ECG Q5	Atrioventricular third-degree bundle branch block
Clinical ECG Q6	Ventricular tachycardia
Clinical ECG Q7	Acute myocardial infarction
Clinical ECG Q8	Normal ECG
Clinical ECG Q9	Ventricular extrasystole
Clinical ECG Q10	Atrial tachycardia

Some Saudi universities had a calculated GPA (Grade Point Average) out of four while others calculated GPA out of five. To allow a precise analysis, all GPAs submitted out of four were converted into a value out of five. Similarly, each participant was scored out of 12 points on their competency level in interpreting common ECG abnormalities. Scores were converted to a maximum of 10, in order to facilitate interpretation and comparison with similar studies. In addition, according to Coll-Badell et al. [12], a score of at least 7.5 was needed to indicate ECG competence. Therefore, a value of 7.5 was used as a cut-off point; any score less than that was labeled as “low competency” and a score equal to or greater than the cut-off point was assumed to be “high competency”.

The survey consisted of questions to investigate any potential confounding factors that might affect the overall perception of the respondent’s competency level, emphasizing the possibility of undertaking specific training experience in reading and interpreting ECGs.

The sample size formula for a single proportion was used to calculate the minimum number of required participants [14]. Assuming the interns in Saudi Arabia having a low ECG interpretation skill to be 53% of the sample proportion, with a 95% confidence interval, and a sample error of 5%, the sample size was estimated to be 383 subjects. Data collectors were assigned from all the medical colleges included in the study and were asked to obtain the number of medical interns in each college along with their gender distribution. These data were used to calculate the strata size out of the whole sample, in order to have a sample composition similar to the universities of Saudi Arabia. Interns were enrolled conveniently according to the distribution of each sub-group. The data was analyzed using Statistical Package for the Social Sciences (SPSS) version 26. Descriptive statistics were used to calculate the mean, median, mode, standard deviation, and frequency. An inference analysis compared the different variables using the chi-square test. A P-value of 0.05 was considered as a cut-off point for the level of significance.

The ethical approval number (KFU-REC-2022-JUN-ETHICS71) has been obtained and approved by the ethical committee of the deanship of scientific research at King Faisal University. Participation in the study was voluntary. The purpose of the study was stated and the expected time was reported. Online consent was obtained prior to filling out the survey.

## Results

A total of 403 accomplished questionnaires were received through Google Forms. Twenty-three respondents were screened out of the questionnaire because they were not medical interns, three respondents refused to participate in the study, and four submitted the form with missing data. Thus, a total of 373 subjects were included in this study.

As shown in Table 2, this study included 373 medical interns from a total of 15 universities across the Eastern, Western, Northern, Southern, and Central regions of Saudi Arabia. Of these, 203 (54.4%) were male and 170 (45.6%) were female. Seventy-six (20.4%) were new interns, and 297 (79.6%) recently completed their internship. Only 93 (24.9%) had finished their cardiology rotation or were taking it at the time of filling out the survey as part of their internship rotations. Further participant statistics are displayed in Table 2.

**Table 2:** Participants’ characteristics (n = 373)

Item		N (%)
Gender		
Male		203 (54.4%)
Female		170 (45.6%)
At what stage are you?		
Started internship recently		76 (20.4%)
Finished internship recently		297 (79.6%)
Have you taken or are you taking cardiology as part of your internship rotations?		
Yes		93 (24.9)
No		280 (75.1)
At which university are you studying?		
King Saud Bin Abdulaziz University (Jeddah)		5 (1.3%)
Jeddah University		12 (3.2%)
Qassim University		34 (9.1%)
King Khalid University		35 (9.4%)
Umm Alqura University		3 (0.8%)
Al Maarefa University		31 (8.3%)
Taibah University		16 (4.3%)
Northern Border University		28 (7.5%)
Imam Mohammad Ibn Saud University		33 (8.8%)
Princess Noura Bint Abdulaziz University		20 (5.4%)
King Abdulaziz University		2 (0.5%)
Imam Abdulrahman bin Faisal University		53 (14.2%)
Aljouf University		31 (8.3%)
King Faisal University		45 (12.1%)
Al Bahah University		25 (6.7%)
Item	Mean	Std. Deviation
Score (out of 10)	5.40	2.25
GPA (out of 5)*	4.10	0.52

The mean of the overall scores of all the participants was 5.40 out of 10 ± 2.25 standard deviation (SD). Moreover, the mean GPA of the participants was 4.10 out of 5.00 (± 0.52 SD). Furthermore, the stage in which the interns were (i.e., the start or end of their internship year), whether they had finished their cardiology rotation as part of their internship, and their GPA were statistically significant to their level of competency in ECG interpretation, as shown in Table 3.

**Table 3:** Relationship between the participants’ characteristics and their ECG interpretation competency level (n = 373)

Item		Competency level			P
		High		Low	
Gender					0.624
Male	51 (25.12%)		152 (74.88%)		
Female	39 (22.94%)		131 (77.06%)		
At what stage are you?*					0.021
Started internship recently	26 (34.21%)		50 (65.79%)		
Finished internship recently	64 (21.55%)		233 (78.45%)		
Have you taken or are taking cardiology as part of your internship rotations?*					0.0214
Yes	18 (19.35%)		75 (80.65%)		
No	72 (25.71%)		208 (74.29%)		
At which university are you studying?					0.000
King Saud Bin Abdulaziz University (Jeddah)	3 (60%)		2 (40%)		
Jeddah University	0 (0%)		12 (100%)		
Qassim University	8 (23.53%)		26 (76.47%)		
King Khalid University	6 (17.14%)		29 (82.86%)		
Umm Alqura University	0 (0%)		3 (100%)		
Al Maarefa University	5 (16.13%)		26 (83.87%)		
Taibah University	5 (31.25%)		11 (68.75%)		
Northern Border University	0 (0%)		28 (100%)		
Imam Mohammad Ibn Saud University	11 (33.33%)		22 (66.67%)		
Princess Noura Bint Abdulaziz University	0 (0%)		20 (100%)		
King Abdulaziz University	0 (0%)		2 (100%)		
Imam Abdulrahman bin Faisal University	33 (62.26%)		20 (37.74%)		
Aljouf University	2 (6.45%)		29 (93.55%)		
King Faisal University	12 (26.67%)		33 (73.33%)		
Al Bahah University	5 (20%)		20 (80%)		
Item	Mean		Std. Deviation		P
Score (out of 10)	5.40		2.25		
GPA (out of 5)*	4.10		0.52		0.002
*Statistically significant (P < 0.05)					

Table 4 shows that 261 (70%) of the participants had not enrolled in an Advanced Cardiac Life Support (ACLS) course, but almost half of the participants, 180 (48.3%), had taken an ECG elective course outside their universities’ curricula. Of those who had taken an ECG course, 71 (19%) had taken the course two to five years ago; 57 (15.3%), less than 12 months ago; 46 (12.3%), one to two years ago; and six (1.6%), more than five years ago. The setting of the course was face-to-face for 112 (30%) of the participants; online for 48 (12.9%); and hybrid (both online and onsite) for only 20 (5.4%).

**Table 4:** Enrollment in extracurricular courses (n = 373) ACLS: Advanced Cardiac Life Support

Item	N (%)
Did you finish the ACLS course?	
Yes	112 (30)
No	261 (70)
Have you ever taken an electrocardiography course apart from that in the university’s curriculum?	
Yes	180 (48.3)
No	193 (51.7)
When did you take the last course? (n = 180)	
Less than a year ago	57 (31.67%)
1–2 years ago	46 (25.56%)
2–5 years ago	71 (39.44%)
More than 5 years ago	6 (3.33%)
How was the course held? (n = 180)	
Online	48 (26.67%)
Face-to-face	112 (62.22%)
Hybrid (face-to-face and online)	20 (11.11%)
How many hours long was the course? (n = 180)	
	137 (76.11%)
10–20	33 (18.33%)
> 20	10 (5.56%)
ACLS; Advanced Cardiac Life Support	

Regarding the number of hours spent in courses, 137 (36.7%) of the participants had courses of less than 10 hours; 33 (8.8%), had 10-20 hours; and only 10 (2.7%), had more than 20 hours. No significant statistical association was found between enrollment in ACLS and ECG courses in general. Nevertheless, the number of learning hours in ECG courses appeared to have a significant relationship with the ECG interpretation competency level, as demonstrated in Table 5.

**Table 5:** Relationship between enrollment in extracurricular courses and the ECG interpretation competency level (n = 373) ACLS: Advanced Cardiac Life Support

Item	Competency level		P
	High	Low	
Did you finish the ACLS course?			0.593
Yes	25 (22.32%)	87 (77.68%)	
No	65 (24.9%)	196 (75.1%)	
Have you ever taken an electrocardiography course apart from that in the university’s curriculum?			0.729
Yes	42 (23.33%)	138 (76.67%)	
No	48 (24.87%)	145 (75.13%)	
When did you take the last course? (n = 180)			0.964
Less than a year ago	14 (24.56%)	43 (75.44%)	
1–2 years ago	10 (21.74%)	36 (78.26%)	
2–5 years ago	17 (23.94%)	54 (76.06%)	
More than 5 years ago	1 (16.67%)	5 (83.33%)	
How was the course held? (n = 180)			0.327
Online	12 (25%)	36 (75%)	
Face-to-face	28 (25%)	84 (75%)	
Hybrid (Face-to-face and online)	2 (10%)	18 (90%)	
How many hours long was the course?* (n = 180)			0.017
< 10	30 (21.9%)	107 (78.1%)	
10–20	6 (18.18%)	27 (81.82%)	
> 20	6 (60%)	4 (40%)	
*Statistically significant (P < 0.05) ACLS; Advanced Cardiac Life Support			

**Figure 1:**
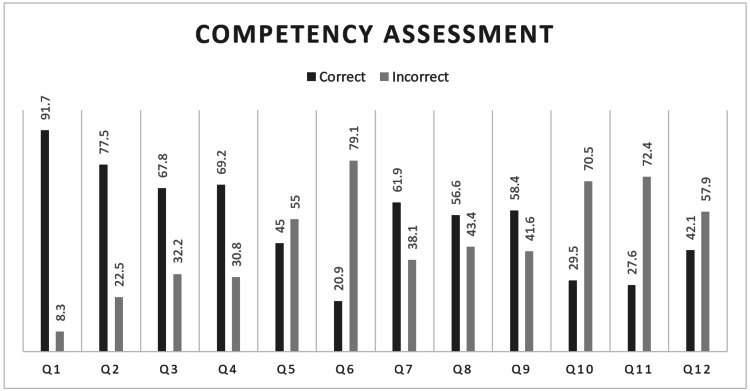
Percentages of correct and incorrect responses to each question

The accuracy of the responses to the competency assessment questionnaire is summarized in Figure 1. The most common misinterpretation of ECG by the participants concerned pathological Q waves, which only 78 (20.9%) of the participants recognized correctly. Other less accurately interpreted ECG patterns were those for ventricular extra-systole, normal ECG, and atrial tachycardia, which were accurately rated by only 27.6%, 29.5%, and 42.1% of the participants, respectively. Almost all the participants (91.7%, 342) widely recognized basic ECG elements, as they correctly determined the normal ECG pattern. The most well-understood ECG pathologies were ventricular fibrillation, atrial fibrillation, and acute myocardial infarction, which were accurately interpreted by 69.2%, 67.8%, and 61.9% of the participants, respectively.

As demonstrated in Table 6, when the participants were asked what could facilitate accurate ECG interpretation by Saudi medical interns, most of them - 214 (57.4%) - chose practical case-based training; 111 (29.8%), extracurricular educational courses; 63 (16.9%), volunteer work in cardiology clinics; and 95 (25.5%), all the stated methods.

**Table 6:** Interns’ perspectives of ECG learning barriers

Item	N (%)
In your opinion, what could facilitate Saudi medical interns’ development of ECG interpretation skills?**	
Practical case-based training in college	214 (57.4)
Extracurricular educational courses	111 (29.8)
Volunteer work in cardiology clinics	63 (16.9)
All of the above	95 (25.5)
Other	15 (4)
In your opinion, what are the limitations of Saudi medical interns in developing ECG interpretation skills?**	
Inadequate training in college	237 (63.5)
Dependence on cardiologist expertise	124 (33.2)
ECG interpretation itself is difficult	155 (41.6)
Lack of resources	65 (17.4)
All of the above	34 (9.1)
Other	18 (4.8)
**Multiple answers are possible.	

As for the drawbacks in developing accurate ECG interpretation skills, most of them - 237 (63.5%) - answered with inadequate training in college; 155 (41.6%), the difficulty of ECG interpretation; 124 (33.2%), dependence on the cardiologist's expertise; 65 (17.4%), lack of resources; and 34 (9.1%), all the stated factors. A few of the participants - 18 (4.8%) - mentioned other limitations, such as few training hospitals and the poor socioeconomic status of undergraduates that could drive them to purchase low-quality or older editions of textbooks or could prevent them from joining excellent training sessions. Some of the participants reported other limitations, such as weak training, lack of small groups teaching ECG, lack of trainers, and repetitive revisions of ECG basics.

## Discussion

Our study showed poor performance of the medical interns in ECG interpretation, which is compatible with the results of a previous study in Iran on the ECG interpretation competency of healthcare providers and students. That study reported low ECG interpretation competency, with an average score of 5.3 ± 2.2 and a top score of 10 [15]. Similarly, Jablonover et al. found the suboptimal performance of graduating medical students in ECG interpretation, with only eight (37%) of 22 key ECGs correctly identified [16]. However, a study at a Peruvian university revealed medium and high levels of knowledge of ECG interpretation among graduating medical interns [17]. This same study demonstrated that a high weighted average is associated with increased competency in ECG interpretation, which is similar to our finding of a positive correlation between the GPA of the participants and their scores in our survey.

Surprisingly, the participants who completed their internship showed a significantly lower score than those who have just begun their internship. Conversely, De Jager et al. concluded that ECG interpretation by recently qualified emergency physicians and emergency residents significantly improved with increasing seniority [18]. On the other hand, a study conducted at Taif University found that seniority had no effect on ECG interpretation [11]. Furthermore, the interns who had been on a cardiology rotation during their internship scored significantly lower than those who had not been trained in a cardiology department. This result is inconsistent with that of a previous study in which repeated practice had a significant impact on ECG interpretation accuracy due to the greater exposure of the subjects during cardiology rotation [19]. This finding does not eliminate the importance of clinical exposure in improving the ECG interpretation, rather than emphasizing the possible role of other unidentified factors. Nevertheless, solely being present on ward rounds or in the emergency unit does not prompt the acquisition of ECG competence. Rather, it should be actively encouraged that during their clinical training, interns analyze and interpret ECGs. This largely self-directed learning endeavor could help with experience-building and reframing ECG knowledge [20].

Participation in ACLS and ECG interpretation courses did not significantly affect the overall performance of the participants, except when the number of hours spent in such courses was factored in. The findings of Apaza-Ramos et al. were similar to ours, which demonstrated that participation in extracurricular courses on ECG interpretation did not significantly affect the overall performance of the participants [17]. In contrast, the findings of this study do not support those of Mobrad et al. of a positive correlation between ACLS courses and the improvement of ECG reading skills [13]. Furthermore, McAloon et al. found that teaching programs can enhance the confidence of undergraduate trainees and their skills in ECG interpretation [21].

In this study, a pathological Q wave was the most incorrectly interpreted ECG pattern, as only 20.9% of the participants recognized it. Mobrad et al. had a similar finding as it was 22% [13]. This emphasizes that detecting chronic myocardial infarction with a pathological Q wave can be challenging for medical interns; and to address this knowledge gap, ongoing ECG interpretation competency training is needed for this context. However, the participants in our study showed good knowledge of ECG basics, as proven by the correctly identified normal ECG pattern by 91.7% of them. Our study supports a prior study that demonstrated a sufficient level of theoretical ECG knowledge among medical students [22].

A vast majority of the participants in this study reported their need for case-based cardiology training. Although all Saudi universities use case-based ECG questions in the pre-clinical and clinical years, educational assessment techniques in Saudi universities must be enhanced to help develop accurate ECG interpretation skills among medical interns. Various studies have evaluated the effects of different instructional methods on ECG training. Sessions, computer-assisted learning, and a newly developed ‘puzzle’ method have all been shown to improve medical students’ ability to interpret ECG [4]. Saudi medical colleges should provide and encourage the uptake of extracurricular educational courses and volunteer work in cardiology clinics. Some of the participants of this study had different recommendations such as understanding the mechanisms behind ECG abnormalities, peer-to-peer courses, and interpretation of real-life ECGs rather than textbook exercises.

The most common drawback of the medical interns in this study was the inadequate ECG training in universities. This finding is consistent with the literature, as previous studies found that only 50% and 75% of junior and senior medical students, respectively, were exposed to ECGs. A distinguishable difference had been found among medical residents, all of whom were exposed to ECGs. This highlights the importance of optimizing medical university curricula toward enhancing clinical exposure and repetitive practice for undergraduate and postgraduate students [21].

We understand the presence of some limitations in this study. First, the number of participating interns in four universities out of 15 didn’t meet the requirement of the strata, which may have led to unequal representation. Additionally, we weren't able to avoid the possibility of repetitive responses, as the accessibility of the survey to the respondents gave them unlimited answer attempts. Lastly, we couldn’t eliminate the possibility of interns sharing answers, browsing the internet, or referring back to textbooks.

We highly recommend enriching medical undergraduates and postgraduates with real-life case-based questions and implementing ECG training more frequently in teaching hospitals. Raupach et al. also demonstrated that peer teaching is an efficient instructional strategy for ECG interpretation. According to their data, small-group, near-peer teaching was at least as effective as lectures by highly qualified electrocardiographers [4]. We further suggest conducting a wider comparative study of the ECG interpretation skills of healthcare professionals and medical interns and students. Finally, conducting an assessment with active surveillance would eliminate possible confounding factors related to distance online assessment.

## Conclusions

This study is the first to evaluate the ECG interpretation proficiency of medical interns at several Saudi universities. According to our findings, most of the medical interns performed below expectations. Despite some engaging in advanced cardiac life support courses, their overall performance did not significantly improve. Most of them identified case-based training as a crucial tactic for their achievement of ECG interpretation proficiency, as they felt that their colleges do not give them enough instructions to enhance their capacity to read and interpret ECGs.
